# Evolutionary Characteristics and Influencing Factors of Public Response Behaviors in Sudden Natural Disasters: Evidence from Typhoon Ragasa in China

**DOI:** 10.3390/bs16101831

**Published:** 2026-10-07

**Authors:** Jiaxin Zhang, Yanyan Wang

**Affiliations:** Faculty of Humanities and Social Sciences, Harbin Institute of Technology, Harbin 150001, China; 26s016011@stu.hit.edu.cn

**Keywords:** public response behaviors, sudden natural disasters, social media, risk perception, social capital, Typhoon Ragasa

## Abstract

Sudden natural disasters pose growing challenges to emergency governance. Understanding public response behaviors is critical to effective risk communication. Social media comments provide valuable data for examining such behaviors during disasters. To investigate the characteristics and influencing factors of public response behaviors, this study used Typhoon Ragasa in 2025 as a case and analyzed 8365 valid comment samples from the Douyin platform. Qualitative coding and text analysis revealed public response behaviors varied across disaster phases. Information behavior predominated during the warning phase, as people relied on information to assess risks and adjust daily routines. Emotional behavior increased during the impact phase, with regional empathy and cross-verification of information. During the recovery phase, responses shifted toward restorative emotional expression and reflection on disaster governance. Action and supportive behaviors remained relatively infrequent. Logistic regression results further showed that the cognitive risk perception index and reply-based conversational engagement were positively associated with information behavior and negatively associated with emotional behavior. Neither was significantly associated with action or supportive behavior. Like-based social endorsement was positively associated with information behavior during the warning phase and emotional behavior during the impact phase. Based on these findings, several recommendations are proposed.

## 1. Introduction

Sudden natural disasters can result in casualties, infrastructure damage, and substantial economic losses. They can also rapidly reshape public information needs, emotional states, and behavioral choices. The effectiveness of disaster governance depends not only on governmental actions, but also on how the public perceives risks and responds to them. Studies of real-world emergencies suggest that individuals may initially respond to the same threat in different ways, including fleeing, investigating, freezing, seeking information, or helping others (Philpot & Levine, 2022). Accordingly, the public can no longer be regarded merely as passive recipients of protection but should also be understood as active participants in risk governance. Consequently, understanding the diversity of public response behaviors during sudden natural disasters is important for improving risk communication and emergency governance.

The rapid development of information technologies and social media has made public responses to disasters increasingly observable through digital activities, extending disaster-related behaviors from offline settings into online spaces (Simon et al., 2015). Disaster communication on social media is not simply a top-down process. Rather, it operates through an information network involving governments, media organizations, social groups, and the general public (Hu et al., 2024; Yudarwati et al., 2022). Government agencies can use social media to disseminate disaster information and conduct risk communication, whereas members of the public can receive and share disaster-related information, comment on events, express concerns, seek reassurance, and establish emotional connections through online interactions (Lambert, 2020). At the same time, digital traces generated through posting, commenting, and sharing provide the data foundation for observing and analyzing public response behaviors during disasters.

Drawing on such digital data, researchers can identify public concerns, emotional expressions, and behavioral characteristics and investigate how they change over the course of a disaster. Research on Hurricane Irma showed that information flows, key communication actors, and widely shared messages differed across the pre-disaster, disaster, and post-disaster phases (Hu et al., 2024). Social media research on Hurricane Florence similarly revealed differences in the topics of concern and emotional responses of different demographic groups (Yuan et al., 2021). In the Chinese context, research on the “7.20” rainstorm in Henan Province identified clear temporal changes in public attention and emotions (Chen et al., 2025), while another study based on Weibo data found that public emotions and response behaviors varied across different stages of the rainstorm event (Wan et al., 2023). These studies indicate that public responses on social media are dynamically adjusted in relation to changing disaster risks, information environments, and real-world conditions. Accordingly, a disaster-process perspective is needed to compare public response behaviors across different stages of an event.

Despite substantial progress, direct comparisons among different types of public response behaviors remain relatively limited. Public responses in disaster contexts are inherently multidimensional. Previous studies have shown that people use social media not only to obtain and share disaster-related information and express emotions, but also to seek or circulate help and rescue information and coordinate relief resources (Li et al., 2024; Liu et al., 2024). A systematic review further demonstrated that public responses during disaster recovery may involve informational and emotional support, social solidarity, donations, and economic assistance (Ogie et al., 2022). However, these forms of response have often been examined separately, with relatively little research directly comparing multiple behavioral types within a unified analytical framework. Distinguishing among different categories of public response behaviors and comparing their characteristics across different disaster phases can provide a more comprehensive understanding of public behaviors during disasters.

Risk perception represents an important factor for explaining such behavioral differences. It reflects individuals’ subjective judgments regarding the likelihood of a hazard and its potential consequences (Lechowska, 2022). Previous research has shown that risk perception is closely related to disaster preparedness and willingness to participate in emergency response, but its relationship with behavior may differ across behavioral types. For example, research conducted in typhoon contexts found that higher levels of risk perception were associated with more active disaster-preparedness behaviors (Ng, 2022), whereas a study of emergency rescue participation among Chinese citizens showed that greater perceived emergency risk was associated with a lower willingness to participate in rescue activities (R. Zhang, 2025). Meanwhile, public responses are also embedded in social relationships and interaction networks. Pre-existing social ties and newly formed mutual-aid networks during disasters can provide important channels through which individuals and communities obtain and mobilize social resources and are closely related to disaster resilience and post-disaster recovery (Uekusa et al., 2022). Social media provides new opportunities to activate these social connections, and bridging and linking social capital have been associated with more active online communication during post-disaster recovery (Page-Tan, 2021). Nevertheless, different dimensions of social capital may relate differently to disaster-response behaviors. In the context of disaster adaptation in China, for example, social networks were negatively associated with farmers’ adaptive behaviors, whereas social trust increased the likelihood of adopting disaster-adaptation measures (Z. Zhang et al., 2023). These findings indicate that both risk perception and different dimensions of social capital may have heterogeneous relationships with different types of public response behaviors. Further comparative investigation is needed to clarify how these factors are associated with different types of response behaviors.

Behavioral research on evacuation shows that emergency outcomes depend on how people make decisions and move in response to danger. At the individual level, reaction time, exit choice and its adaptation, movement speed, and bottleneck behavior can alter evacuation efficiency (Haghani & Yazdani, 2024a, 2024b). At the group level, crowd composition and changes in social-group behavior can also affect evacuation efficiency (Haghani & Yazdani, 2025). These findings distinguish participation from performance: the consequences of action depend on timing, physical conditions, and coordination. They provide a basis for considering the feasibility of action in disaster response.

Public interaction during disasters may also vary substantially across platform contexts. Previous research has shown that users’ perceptions of platform affordances influence how information is received and disseminated and that patterns of participation differ across social media platforms (Dvir-Gvirsman et al., 2024). In earthquake contexts, Twitter and Reddit users have exhibited marked differences in topics of concern and emotional expression, while much of the existing disaster-related social media literature has focused on platforms such as Twitter and Facebook, leaving cross-platform research comparatively underdeveloped (Ruan et al., 2022). The growth of short-video platforms such as TikTok has created a new environment for disaster communication through audiovisual forms of interaction, yet their implications for public participation and emotional responses remain insufficiently understood (Liu et al., 2024). Notably, although Douyin and TikTok share many functional characteristics and platform affordances, they operate in different market and governance environments (Kaye et al., 2021). It remains uncertain whether findings derived from other social media contexts can be directly generalized to Chinese short-video platforms. Examining multiple types of public response behavior throughout the disaster process on a Chinese short-video platform can provide additional evidence on digital disaster communication and help assess the applicability of previously identified behavioral patterns and relationships in this platform context.

Against this background, this study uses Typhoon Ragasa in 2025 as a case and analyzes related comments collected from Douyin to address two research questions: RQ1: How do different types of public response behaviors vary across different phases of a typhoon disaster? RQ2: How are comment-level cognitive risk perception and platform-based interaction associated with different types of public response behaviors? To address these questions, this study first develops a theoretical framework and corresponding hypotheses based on previous research, classifying public response behaviors into four categories: information, emotional, action and supportive behavior. The typhoon process is further divided into three phases: warning, impact, and recovery. Second, qualitative coding and text analysis are used to identify different types of public response behavior and compare their characteristics across disaster phases. Finally, indicators of cognitive risk perception and platform-based interaction are constructed, and logistic regression models are employed to examine their statistical associations with the four types of public response behavior and to test the proposed hypotheses.

## 2. Theoretical Framework and Research Hypotheses

### 2.1. Theoretical Framework

Public response behaviors during sudden natural disasters refer to behavioral reactions formed through information processing, risk judgment, and social interaction under specific risk situations. Weber’s theory of social action emphasizes that social action is endowed with subjective meaning and oriented toward the behavior of others (Weber, 1978). In disaster situations, people may use social media to ask for and share disaster-related information, express concerns or encouragement, take protective actions, and provide help to others. These behaviors reflect how individuals understand disaster risks and interact with other social actors.

Although previous studies have not reached a completely consistent definition of public response behaviors, existing research has demonstrated their multidimensional characteristics. In social media environments during natural disasters, people may release emergency information, update disaster conditions, coordinate resources, and express emotions (Li et al., 2024). A systematic review also showed that social media can support various disaster-related functions, including information support, emotional support, social connection, and donations or economic assistance (Ogie et al., 2022). Based on previous studies and the characteristics of disaster-related social media interactions, this study defines public response behaviors as a series of behavioral reactions with social meanings and psychological motivations expressed by the public through social media platforms during sudden natural disasters. In this study, public response behaviors are classified into four categories as follows:

- Information behavior

Refers to behaviors through which individuals obtain, share, comment on, or verify disaster-related information through social media. These behaviors include reposting official warning information, sharing disaster-related knowledge, verifying information, and reporting on-site situations.

- Emotional behavior

Refers to the expression of emotional reactions and psychological support related to disasters through social media. It includes expressing fear and concern, showing sympathy for affected people, offering encouragement, and expressing support for rescue workers.

- Action behavior

Refers to specific response activities taken by individuals in online or offline environments. These behaviors include preparing emergency supplies, adopting protective measures, participating in volunteer rescue activities and assisting evacuation.

- Supportive behavior

Refers to providing resources or services to affected groups through material or virtual forms. It includes donations, providing temporary accommodation, organizing fundraising activities, and offering professional assistance.

To explain differences among the four response categories, this study focuses on risk perception and social capital as two complementary factors. Risk perception captures how individuals appraise the severity, uncertainty, and potential consequences of a disaster. Such appraisals provide a cognitive basis for information processing, emotional reactions, and protective decisions, although their behavioral implications may differ across response types and situations (Lindell & Perry, 2012; Ng, 2022; R. Zhang, 2025). Social capital captures the relational conditions through which people obtain information, evaluate credible communication, interact with others, and access support or resources during disasters (Gultom, 2016; Page-Tan, 2021; Uekusa et al., 2022). Risk perception therefore represents an appraisal-related factor, whereas social capital represents a relational-resource factor. Together, they provide two complementary explanations for variation in public response behaviors.

The relevance of these factors may differ across behavioral categories because the four responses involve different functions and conditions. Information behavior can help reduce uncertainty through information seeking, sharing, and verification, while emotional behavior provides an immediate means of expressing threat appraisal, concern, sympathy, or encouragement. By comparison, action behavior requires that risk appraisal be translated into feasible protective or participatory activities. Supportive behavior further depends on the recognition of others’ needs and the availability of relationships, resources, and opportunities through which assistance can be provided. Accordingly, risk perception may be associated with the four behaviors through informational, affective, protective, and helping motivations, whereas social capital may support them through information access, relational recognition, coordination, and resource mobilization. Information and emotional responses can generally be expressed directly in social media comments, while the enactment of action and support may face stronger practical constraints.

These mechanisms are considered within the changing context of disaster phases. Previous research has documented differences in information flows, social media use, and public emotional and behavioral expressions across disaster phases (Takahashi et al., 2015; Wan et al., 2023; Hu et al., 2024). During the warning phase, uncertainty reduction and preparedness may make information access and credible communication particularly relevant. During the impact phase, immediate threat appraisal, real-time verification, and emotional support may become more salient, while hazardous conditions may restrict physical participation. During recovery, information about service restoration, identification of assistance needs, and access to organizational and relational resources may become increasingly relevant (Ogie et al., 2022). Disaster phase is therefore treated as a contextual condition that may alter the salience and feasibility of different response mechanisms.

The three theoretical perspectives perform distinct roles within this framework. Social action theory provides the conceptual basis for interpreting public responses as meaningful and other-oriented behaviors; the four behavioral categories are informed by disaster communication research rather than derived directly from Weber’s typology (Weber, 1978; Li et al., 2024; Ogie et al., 2022). Risk society theory explains how disaster threats acquire social meaning through institutional knowledge, media communication, and public interpretation, while risk perception represents the more proximal appraisal factor examined in the empirical analysis (Beck, 1992). Social capital theory explains how networks, trust, and relational resources may facilitate information access, social recognition, coordination, and assistance (Bourdieu, 1986; Coleman, 1988; Portes, 1998; Putnam, 2000). The resulting framework therefore connects disaster-phase context with an appraisal-related pathway and a relational-resource pathway to explain differences among information, emotional, action, and supportive behaviors.

### 2.2. Research Hypotheses

#### 2.2.1. Risk Perception

Risk perception refers to individuals’ subjective appraisals of the likelihood, severity, uncertainty, and potential consequences of a hazard rather than a simple reflection of objective risk conditions. In natural-disaster settings, these appraisals provide a basis for deciding whether further attention or a response is required (Slovic, 1987; Lechowska, 2022).

Risk perception may be associated with the four behavioral categories through different mechanisms. For information behavior, a perceived gap between what individuals know and what they believe they need to know creates information insufficiency. This insufficiency can motivate the search, comparison, verification, and sharing of information about the location, timing, severity, and possible consequences of a threat. Evidence from the Risk Information Seeking and Processing model and flood-risk communication research supports this uncertainty-reduction pathway (Yang et al., 2014; Kellens et al., 2013).

For emotional behavior, threat appraisal can activate affective responses such as fear, concern, powerlessness, or sympathy. Cognitive judgments concerning possible harm and affective reactions to that harm can operate jointly, while comment sections provide an immediate and relatively low-cost setting in which these reactions can be expressed (Loewenstein et al., 2001; Terpstra, 2011).

For action behavior, the recognition of possible harm can make preparedness and self-protection more salient. However, risk perception alone is not sufficient to produce protective action. Its translation into action also depends on response efficacy, perceived capacity, feasible behavioral options, and situational resources. Reviews of flood mitigation behavior consequently report that the empirical relationship between risk perception and protective behavior is often conditional rather than uniformly positive (Lindell & Perry, 2012; Bubeck et al., 2012; Ng, 2022).

For supportive behavior, the relevant appraisal shifts from personal exposure to the consequences faced by other people. Recognizing others’ losses and unmet needs may elicit sympathy and motivate the provision of encouragement, information, services, or material assistance. Disaster-donation research nevertheless indicates that helping depends on how victims and their needs are appraised, rather than on perceived disaster risk alone. Immediate personal danger may instead direct attention and resources toward self-protection (Zagefka et al., 2011; R. Zhang, 2025).

Therefore, the following hypotheses are proposed:

**H1a.**  *Risk perception is positively associated with public information behavior.*

**H1b.**  *Risk perception is positively associated with public emotional behavior.*

**H1c.**  *Risk perception is positively associated with public action behavior.*

**H1d.**  *Risk perception is positively associated with public supportive behavior.*

These hypotheses express broad positive theoretical expectations, but they do not imply that the associations will be equally strong across response categories or disaster phases. In the empirical analysis, risk perception is operationalized using a text-based cognitive risk perception index at the comment level, capturing three dimensions—occurrence likelihood and exposure, susceptibility and vulnerability, and consequence and severity—as detailed in Section 3.3.2.

#### 2.2.2. Social Capital

Social capital refers to resources embedded in social relationships and made accessible through networks, trust, reciprocity, and shared norms. In disaster contexts, bonding, bridging, and linking relationships may provide different resources: bonding ties connect close relations, bridging ties connect otherwise separate groups, and linking ties provide access to institutions and organizations. Therefore, these dimensions should not be assumed to influence all response categories through the same process (Bourdieu, 1986; Coleman, 1988; Aldrich & Meyer, 2015).

For information behavior, bonding ties can provide rapid local information, bridging ties can broaden the range of available sources, and linking ties can connect individuals with institutional warnings and official guidance. Trust can increase the perceived credibility of communicated information, while interaction among multiple contacts creates opportunities for questioning and verification. Disaster-communication and social-messaging research supports the role of trusted networks in accessing and mobilizing information during emergencies (Gultom, 2016; Chu & Yang, 2020).

For emotional behavior, bonding relationships can provide recognition, reassurance, and a sense of belonging, thereby creating opportunities for emotional exchange. Evidence from Hurricane Katrina and post-disaster mental-health research shows that relational resources may provide emotional and psychosocial support following disaster exposure. This mechanism concerns opportunities for expressing and responding to emotion, rather than an assumption that social capital necessarily intensifies negative emotion (Hawkins & Maurer, 2010; Wind & Komproe, 2012).

For action behavior, bridging and linking relationships can transmit trusted preparedness advice and connect individuals with organizations, evacuation resources, and opportunities for participation. Trust and reciprocity may also reduce the coordination costs involved in collective preparation. Empirical research has linked social capital with disaster preparedness, although the realization of protective action continues to depend on physical capacity, safe access, and feasible behavioral options (Reininger et al., 2013).

For supportive behavior, networks can help identify recipients, communicate needs, pool resources, and coordinate assistance. Bonding ties may facilitate immediate help among close relations, whereas bridging and linking ties may provide access to more diverse resources and institutional support. These benefits are not distributed equally, because individuals and communities with limited network resources may be less able to obtain or mobilize assistance (Uekusa et al., 2022; Elliott et al., 2010). Therefore, the following hypotheses are proposed:

**H2a.**  *Social capital is positively associated with public information behavior.*

**H2b.**  *Social capital is positively associated with public emotional behavior.*

**H2c.**  *Social capital is positively associated with public action behavior.*

**H2d.**  *Social capital is positively associated with public supportive behavior.*

For theoretical purposes, H2a–H2d are formulated at the level of the broader social capital construct. However, the empirical analysis uses platform-interaction indicators rather than direct measures of social networks or social trust. The findings are therefore interpreted as indicator-level evidence consistent or inconsistent with the theoretical expectations, rather than definitive tests of social capital itself.

**Figure 1:**
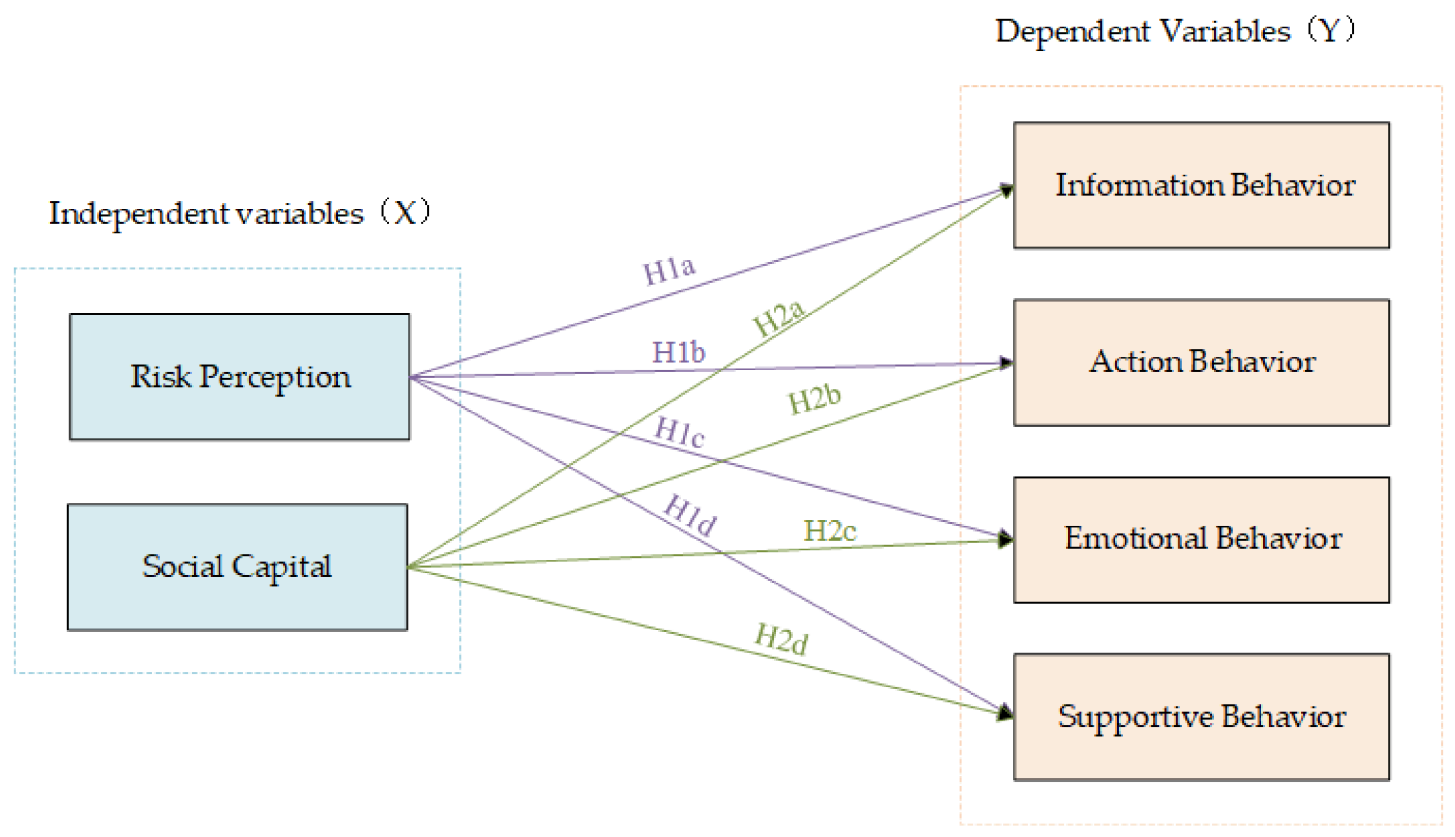
Research hypotheses.

At the empirical level, these theoretical perspectives inform the interpretation of two observable platform-interaction indicators: reply-based conversational engagement and the like-based social endorsement signal. Their definitions and operationalization are presented in Section 3.3.3. Figure 1 presents the research framework and hypotheses proposed in this study.

## 3. Materials and Methods

### 3.1. Event Description

This study selected Typhoon Ragasa in 2025 as a case of a sudden natural disaster. Typhoon Ragasa formed over the western Pacific Ocean on 18 September 2025 and developed into a super typhoon on 21 September. It made landfall near Hailing Island, Yangjiang City, Guangdong Province, China, at approximately 17:00 on 24 September as a typhoon-level storm. The typhoon brought strong winds and heavy rainfall, which caused considerable impacts on public daily life. During the typhoon event, meteorological agencies, emergency management departments, and local governments released warning information, disaster updates, and emergency response measures through social media platforms such as Douyin. Meanwhile, many users discussed the disaster situation and shared their experiences through comments. These online interactions provide valuable data for examining public response behaviors throughout the disaster process.

Previous studies have shown that information sharing, online interaction, and social media use may change during different disaster periods (Takahashi et al., 2015). Therefore, this study divided the development of Typhoon Ragasa into three phases: the warning phase, impact phase, and recovery phase. Based on meteorological warnings, emergency response processes, typhoon movement, and post-disaster recovery progress, the warning phase was defined as 00:00 on 21 September to 23:59 on 23 September 2025. The impact phase was defined as 00:00 on 24 September to 23:59 on 25 September 2025. The recovery phase started from 00:00 on 26 September 2025 and continued until the end of data collection.

### 3.2. Data Collection

The research data were collected from publicly available user comments under videos related to Typhoon Ragasa on Douyin. Data collection was completed on 8 December 2025, covering the period from 21 September 2025 at 00:00 to 8 December 2025 at 20:00. A data collection program was developed using Python 3.13.7 to obtain comment texts and interaction information.

This study employed purposive sampling. The sampling frame was restricted to institutional accounts that regularly disseminated disaster-related information. This sampling strategy was adopted to examine public responses to identifiable and relatively credible disaster information across national, governmental, and local communication contexts. It was not intended to produce a statistically representative sample of all Douyin users or all typhoon-related discussions on the platform.

Using ‘Typhoon Ragasa’ as the search term, all videos concerning Typhoon Ragasa posted by each selected account during the study period were included. For every eligible video, the collection program retrieved all comments displayed across the finite pages accessible on Douyin at the time of collection, including second-level replies. Because comment visibility is shaped by platform ranking, moderation, deletion, and pagination, the resulting dataset represents all publicly accessible comments retrievable from those displayed pages. To ensure source credibility and capture diverse institutional communication contexts, this study selected videos from three types of accounts. First, national-level authoritative media outlets were included, such as CCTV News, CCTV.com, People’s Daily, People.cn, Xinwen Lianbo (News Broadcast), and ThePaper.cn. These outlets provide timely and credible information with broad public influence. Second, government authorities were selected, including the Ministry of Emergency Management (MEM) and the National Meteorological Center (NMC), which mainly release official meteorological information, emergency response instructions, and disaster updates. Third, local media and emergency organizations were included, such as Yangjiang Observer and Guangdong Fire and Rescue, which focus on frontline information, local impacts, and public services in the typhoon-affected areas. These accounts provided complementary information on national warnings, emergency-response measures, local disaster impacts, and post-disaster recovery. A total of 10 Douyin accounts were included. The collected data contained account information, video ID, user nickname, user ID, user location, posting time, comment text, comment likes, and comment replies. Only publicly available information displayed on the platform was collected. Private messages, restricted profile information, and other non-public data were not included.

The initial dataset contained 10,464 comments from 91 videos. After data cleaning and standardization, including removing empty and duplicated comments, correcting format problems, handling missing information, and excluding abnormal records, the final dataset contained 8365 valid comments from 88 unique videos. The analytic sample included 4557 comments under national-level authoritative media outlets, 891 under government authorities, and 2917 under local media and emergency organizations. By disaster phase, 1472 comments were collected during the warning phase, 5139 during the impact phase, and 1754 during the recovery phase. For data management, each comment was assigned a unique identification code according to disaster phase, video order, and comment order. YJ, FS, and HF represent the warning phase, impact phase, and recovery phase, respectively, while V and C represent video and comment numbers. For example, YJ-V0001-C0001 represents the first comment under the first video during the warning phase. The comment was the unit of analysis. Accordingly, all reported associations are interpreted at the comment level.

### 3.3. Variable Measurement

#### 3.3.1. Public Response Behaviors

The four types of public response behaviors were used as dependent variables in the regression models. After the three-stage coding process was completed, the comment-level coding matrix was exported from NVivo 15. Information behavior, emotional behavior, action behavior, and supportive behavior were transformed into binary variables. When a comment contained a specific type of behavior, the variable was coded as 1. When the behavior did not appear, the variable was coded as 0. One comment could contain more than one type of behavior.

#### 3.3.2. Cognitive Risk Perception Index

Risk perception is conceptualized in this study as an individual appraisal of disaster-related threats. In the empirical analysis, however, it is not measured through self-reported perceptions but operationalized from observable textual expressions in social media comments. To maintain consistency with the theoretical construct while avoiding operational overlap with emotional behavior, this study constructs a text-based cognitive risk perception index at the comment level. The index captures three dimensions of cognitive risk expression: occurrence likelihood and exposure, susceptibility and vulnerability, and consequence and severity.

Natural language and social-media data have increasingly been used to identify observable expressions related to public risk perception. Word use and semantic relationships can provide information about how individuals represent and evaluate risks (Bhatia, 2019), while studies of public-health and extreme-weather events have demonstrated the value of social-media texts for examining risk-related attention, topics, and emotional reactions (Dyer & Kolic, 2020; Qiu et al., 2025; R. Zhang & Chen, 2025). These studies provide a general basis for using textual expressions as empirical indicators.

Risk perception is a multidimensional construct involving both cognitive judgments and affective reactions. The risk-as-feelings framework distinguishes emotional responses from cognitive evaluations of the likelihood and consequences of a hazard (Loewenstein et al., 2001). Related research further differentiates deliberative, affective, and experiential components of perceived risk (Ferrer & Klein, 2015; Ferrer et al., 2016), as well as probabilistic, consequential, and susceptibility-related dimensions (Wilson et al., 2019; Walpole & Wilson, 2021). Because emotional behavior is one of the dependent variables in this study, including fear, anxiety, negative sentiment, or wishes for safety in the explanatory variable would create operational overlap. The empirical measure was therefore restricted to observable cognitive expressions related to risk perception and is referred to as the cognitive risk perception index.

Based on these theoretical distinctions and the characteristics of typhoon-related communication, cognitive risk expressions were classified into three dimensions. The first dimension, occurrence likelihood and exposure ($RP_{Li}$), captures expressions concerning whether, when, or where the typhoon may occur or affect a particular area. It includes references to forecasts, landfall, movement paths, affected areas, warning levels, and event frequency. The second dimension, susceptibility and vulnerability ($RP_{Si}$), captures expressions indicating that particular people, locations, buildings, or infrastructure may be especially exposed to harm or may have insufficient capacity to withstand the hazard. It includes references to low-lying and coastal areas, vulnerable buildings, inadequate drainage, and limited wind or flood resistance. The third dimension, consequence and severity ($RP_{Ci}$), captures expressions concerning the intensity and potential or realized consequences of the typhoon, including strong winds and rainfall, flooding, physical damage, casualties, and disruptions to electricity, transportation, and other public services. Hazard-intensity descriptions were treated as partial cognitive cues to severity.

A domain-specific lexicon was developed through a theory-guided and corpus-informed procedure. First, seed terms were selected according to the definitions of the three dimensions. Second, the seed terms were expanded using semantically related expressions, typhoon-specific terminology, and expressions identified in the comment corpus. Candidate terms were examined in their original comment contexts to identify ambiguous or non-risk uses. Third, exclusion terms and contextual rules were established to remove pure emotional expressions, blessings and wishes, standalone response actions, policy enumerations, account-login contexts, fictional or entertainment contexts, and common idiomatic uses. Consequently, expressions of fear, anxiety, sadness, prayer, and wishes for safety did not contribute to the index. Evacuation, rescue, donation, and preparedness expressions were also excluded when they occurred without an identifiable cognitive reference to the hazard. The final matching list contained 335 dimension-specific terms or regular-expression patterns: 111 for occurrence likelihood and exposure, 52 for susceptibility and vulnerability, and 172 for consequence and severity.

Before matching, the comment texts were normalized using Unicode NFKC normalization, converted to lowercase where applicable, and stripped of whitespace. Lexicon matching followed a longest-match and non-overlapping rule within each dimension. This prevented a longer expression, such as “building collapse”, from being counted again through an overlapping shorter expression such as “collapse”. Ambiguous expressions were evaluated using the surrounding 18-character context. These rules distinguished, for example, typhoon landfall from account login, vulnerable infrastructure from general statements about human fragility, disaster-related power outages from policy enumerations, and coastal vulnerability from tourism or leisure references.

Questions, negated expressions, and descriptions of realized disaster consequences were retained when they contained an identifiable cognitive reference. For example, a question about whether electricity would be interrupted still expresses consideration of a possible disaster consequence. These expressions were not assigned positive or negative directional weights because the index captures the presence and dimensional coverage of cognitive risk content rather than whether the commenter judged the risk to be high or low.

For each comment, the three dimensions were assigned values separately. A dimension was assigned a value of 1 when at least one valid expression belonging to that dimension was identified and 0 otherwise. A comment could be assigned a value of 1 for more than one dimension. Binary dimension indicators were used in the main analysis to prevent longer comments or repeated use of the same expression from disproportionately increasing the index. The three dimensions were assigned equal weights, and the unstandardized cognitive risk perception index for comment *i* was calculated using Equation (1).

(1) $$RP_{i}^{raw}=RP_{Li}+RP_{Si}+RP_{Ci}$$

where $RP_{Li}$, $RP_{Si}$ and $RP_{Ci}$ denote the assigned values for occurrence likelihood and exposure, susceptibility and vulnerability, and consequence and severity, respectively. Accordingly, $RP_{i}^{raw}$ ranged from 0 to 3. Dimension-specific match counts and character-normalized densities were retained as auxiliary indicators for measurement auditing and sensitivity analysis but were not used to construct the principal regression variable.

To facilitate comparison of effect sizes across the explanatory variables, the raw index was standardized across the full sample using Equation (2).

(2) $$RP_{i}=\frac{RP_{i}^{raw}-\bar{RP^{raw}}}{SD(RP^{raw})}$$

where $\bar{RP^{raw}}$ and $SD(RP^{raw})$ denote the sample mean and standard deviation of the unstandardized index, respectively. The standardized $RP_{i}$ was used in the logistic regression models. Its odds ratio therefore represents the change in the odds of a response behavior associated with a one-standard-deviation increase in the number of cognitive risk perception dimensions expressed in a comment. The cognitive risk perception index can be interpreted as a text-based operational indicator. A higher value indicates that a comment contains expressions covering more of the three cognitive dimensions.

#### 3.3.3. Platform-Interaction Indicators

Social capital provides the theoretical perspective for understanding the relational and interactional context of public responses. Social capital has multiple dimensions. The structural dimension focuses on network connections among social actors, whereas the relational dimension involves trust, reciprocity, and relationship quality. In social media environments, social relationships are not only represented by static connections such as friendships or follows but also leave digital traces through observable interactions, including replies, comments, and likes. Accordingly, this study draws on social networks and social trust as theoretical reference points for interpreting two forms of platform interaction: conversational engagement and social endorsement signals. These interaction indicators capture specific observable manifestations potentially related to social capital processes.

On the one hand, the social-network perspective provides a basis for examining conversational engagement. Replying represents a directed interaction centered on a specific comment, in which other users continue information exchange around the original comment, reflecting the interactive connections established through that comment. Previous studies have shown that directed communication behaviors, such as commenting, contribute to relationship maintenance and the formation of social capital (Ellison et al., 2014). Compared with click-based interactions, textual comments usually involve greater communicative investment (Burke & Kraut, 2016), and replies have been examined as manifestations of interactivity in online discussions (Ziegele et al., 2014). Therefore, this study used the number of replies received by each comment to represent the extent of conversational engagement generated around that comment and defined the transformed measure as reply-based conversational engagement.

On the other hand, the social-trust perspective draws attention to recognition, reciprocity, and relational feedback within online interactions. Likes do not represent general social trust as measured in traditional surveys; rather, they reflect low-cost social recognition, attention, and relational feedback. Previous studies have regarded likes as social media interactions associated with relationship maintenance and reciprocity. Research has shown that liking behavior is closely related to relational closeness, reciprocity, and social grooming (Stsiampkouskaya et al., 2023). Likes may also communicate approval or support, although their meanings depend on the interaction context (Hayes et al., 2016). Platform affordances and interaction practices can be associated with social capital processes (L. Zhang & Jung, 2023). Therefore, considering the interaction mechanisms of Douyin, this study used the number of likes received by each comment to capture the volume of visible social endorsement signals. The log-transformed measure was termed the like-based social endorsement.

To reduce the influence of extremely high values on regression estimation, the numbers of comment replies and likes were transformed using natural logarithms. These transformed indicators were included as platform-interaction variables informed by the social capital perspective. The specific calculations are presented in Equations (3) and (4).

(3) $${CE}_{i}=\log(a_{i}+1)$$

(4) $${SE}_{i}=\log(b_{i}+1)$$

where $a_{i}$ is the number of replies received by the *i*th comment, ${CE}_{i}$ denotes its reply-based conversational engagement. $b_{i}$ is the number of likes received by the *i*th comment, and ${SE}_{i}$ denotes the like-based social endorsement associated with that comment.

#### 3.3.4. Covariates

Disaster phase was assigned from each comment’s posting timestamp, using the event windows defined in Section 3.1: the warning phase (21–23 September 2025), impact phase (24–25 September 2025), and recovery phase (26 September–8 December 2025 at 20:00). Thus, phase referred to when the comment was posted, rather than when its parent video was published. Phase entered the regression models as a three-category factor, with the warning phase as the reference category.

Account type was assigned according to the institutional account that published the video under which a comment appeared. The three categories were national-level authoritative media outlets, government authorities, and local media and emergency organizations, as defined in Section 3.2. National-level authoritative media outlets served as the reference category. The platform-displayed posting location associated with each comment was coded as Guangdong (1) or non-Guangdong (0). This location label was not treated as a verified measure of residence or direct exposure to the typhoon.

Comment exposure duration was the elapsed time in days, including fractions of a day, between each comment’s posting timestamp and the common data-collection cutoff of 8 December 2025 at 20:00. It was standardized using the mean and standard deviation of the analytic sample before regression.

### 3.4. Data Analysis Methods

To identify public response behaviors, this study used NVivo 15 to conduct theory-informed qualitative content analysis, combining deductive categorization with inductive thematic coding (Hsieh & Shannon, 2005; Fereday & Muir-Cochrane, 2006). The four broad behavioral categories were informed by previous research, while specific concepts and subthemes were identified from the comments. Drawing on the staged coding logic of grounded theory, the analysis proceeded through initial concept identification, category integration, and thematic classification. These procedures were used to organize and classify behavioral expressions.

During initial coding, each comment was treated as the unit of analysis. Specific concepts and expressions related to public response behaviors were identified from the comment texts, and semantically similar concepts were grouped into initial categories. The warning, impact, and recovery phases yielded 46, 72, and 59 initial categories, respectively. During category integration, the initial categories were compared and organized according to their semantic relationships and behavioral characteristics. This process yielded 11, 24, and 16 broader categories for the warning, impact, and recovery phases, respectively. During thematic classification, these categories were organized within the four literature-informed behavioral domains: information behavior, emotional behavior, action behavior, and supportive behavior. The specific concepts and subthemes within these domains were developed inductively from the comments. This process combined a theory-informed classification framework with data-driven identification of phase-specific expressions.

The individual social media comment was the unit of analysis. Using a standardized coding manual, one coder classified all 8365 comments for the presence or absence of four non-mutually exclusive categories: information, emotional response, action, and supportive behavior. The initial coding, completed in March 2026, was used for all substantive analyses. The same coder recoded all comments in September 2026 to assess intra-coder reliability over time. Cohen’s kappa was 0.702 for information, 0.733 for emotional response, 0.744 for action, and 0.884 for supportive behavior, indicating generally good consistency between the two coding rounds. Because only one coder was involved, inter-coder reliability could not be assessed. Statistical analyses were conducted in R 4.4.2. First, descriptive statistics were used to examine the occurrence proportions of the four public response behaviors. Then, four comment-level binary logistic regression models were estimated for information, emotional, action, and supportive behavior. The focal predictors were the cognitive risk perception index, reply-based conversational engagement, and like-based social endorsement. Each model simultaneously included disaster phase, account type, platform-displayed posting location, and standardized comment exposure duration as covariates. The warning phase and national-level authoritative media outlets were the reference categories. All four continuous predictors were standardized across the analytic sample.

Primary inference was based on HC1 cluster-robust standard errors at the video level (88 clusters), with two-way clustering by video and user conducted as a sensitivity analysis. Overall model fit was summarized using likelihood-ratio tests, AIC, and McFadden’s pseudo-R^2^, whereas predictor-level inference was based on cluster-robust Wald tests. Models omitting exposure duration were fitted to assess sensitivity to its strong alignment with posting phase. Because action and supportive behaviors were rare, Firth logistic regression with intercept correction was also fitted, using profile penalized-likelihood confidence intervals and *p* values. Out-of-sample performance was evaluated using 10-fold cross-validation grouped by video; calibration was summarized by Brier score, calibration intercept, and slope, and discrimination by AUC. All tests were two-sided, with *p* < 0.05 considered statistically significant.

Because comments were nested within videos and videos within accounts, additional generalized linear mixed models were estimated for information and emotional behavior as sensitivity analyses. These models retained the same fixed effects as the primary models and included random intercepts for account and video. Action and supportive behavior were not fitted with multilevel models because their event counts (73 and 88, respectively) were insufficient for stable estimation of additional variance components. The multilevel models were used to assess the robustness of the common-outcome findings.

## 4. Results

### 4.1. Evolutionary Characteristics of Public Response Behaviors

**Figure 2:**
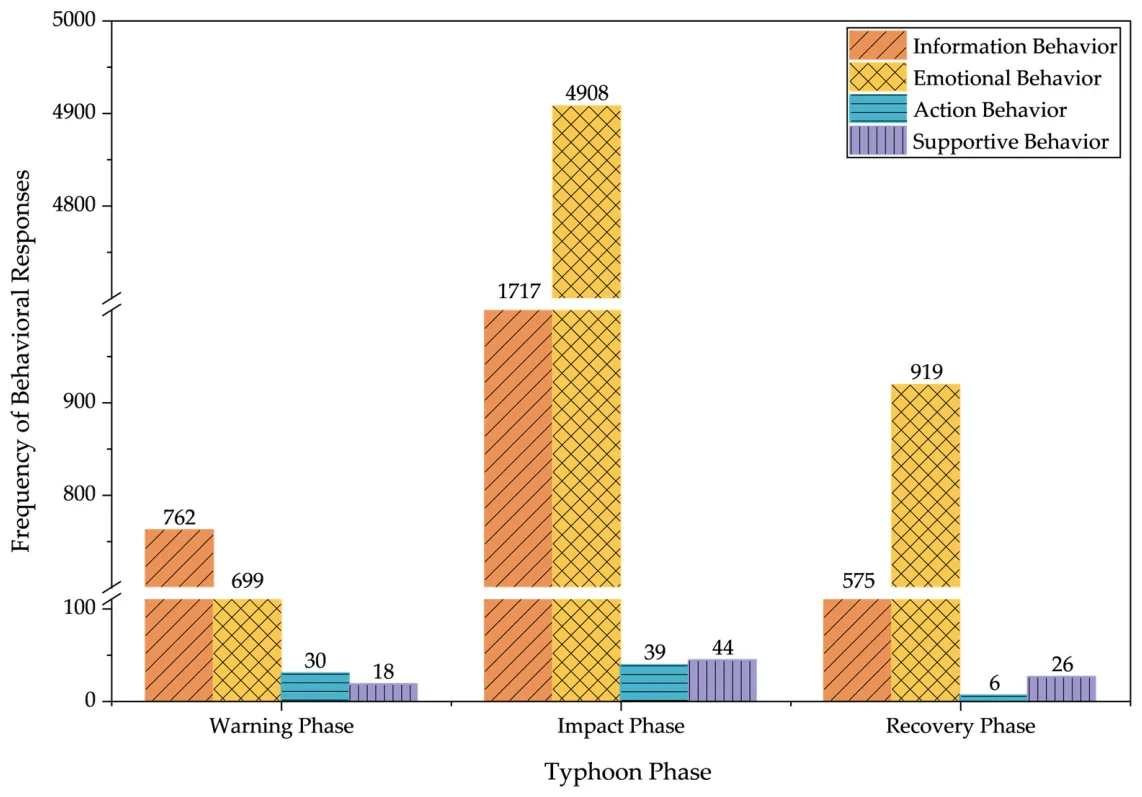
Frequency distributions of public response behaviors across the phases of Typhoon Ragasa.

The frequency distributions of the four public response behaviors based on the number of coded references are shown in Figure 2. Given the substantial differences in frequency across behavioral categories, a broken-axis scale was used to improve visual comparability. Because a single comment could be coded into more than one behavioral category, the total number of coded behavioral references exceeds the number of comments. This coding structure does not affect the analysis of phase-specific patterns across the four behavioral categories.

Emotional behavior was the most frequently observed response throughout the entire course of Typhoon Ragasa, followed by information behavior, whereas action and supportive behaviors occurred relatively infrequently (Figure 2). Clear phase-specific differences were also observed in the distributions of the four behavioral categories. Information behavior was most prominent during the warning phase, emotional behavior reached its highest frequency during the impact phase, and emotional behavior remained dominant during the recovery phase. The proportion of supportive behavior increased to some extent during recovery, while information behavior continued to be observed.

The comment-level distribution in Table 1 complements the NVivo coding-reference frequencies shown in Figure 2. Emotional behavior was the most common category overall (51.88%), followed by information behavior (34.93%), whereas action (0.87%) and supportive behavior (1.05%) were rare. Information behavior was most prevalent during the warning phase (48.85%), while emotional behavior was most prevalent during the impact phase (54.58%).

#### 4.1.1. Public Response Behaviors During the Warning Phase

During the warning phase, public responses were predominantly information-oriented and driven by efforts to reduce or avoid disaster-related risks. Frequently occurring questions such as “When will it make landfall?”, “How strong will the winds be?”, and “Will work be suspended?” indicated that users actively sought disaster-related information to reduce uncertainty surrounding the typhoon risk. In addition to expressing personal concern, some users proactively provided protective advice and safety reminders. Representative comments included: “Those in low-lying areas or near the mountains should evacuate if necessary and return after the typhoon has passed” (YJ-V0005-C0061); “Take proper precautions—this kind of severe typhoon is really powerful” (YJ-V0009-C0014); and “Follow the weather forecast, do not go outside, and stay safe, everyone!” (YJ-V0009-C0352). These comments indicate that, during the warning phase, users acted not only as recipients of warning information but also as contributors to its supplementation and redistribution. The comment section thus functioned, to some extent, as a space for disseminating disaster-prevention knowledge and risk-related reminders.

The qualitative coding further revealed a distinct pattern of negotiation over everyday arrangements during the warning phase. Users discussed whether workplaces and schools would close and whether normal travel would remain possible, as illustrated by comments such as “The boss says it’s fine—we’re working!” (YJ-V0009-C0036), “So there’s no suspension of work for us” (YJ-V0016-C0041), and “Go tell that to my boss” (YJ-V0016-C0066). These comments show that public attention extended beyond the typhoon itself to its potential consequences for work, education, and family life. Disaster warnings therefore became embedded in everyday decision-making and were closely connected to work arrangements, school routines, and individual responsibilities.

Alongside the high frequency of information behavior, emotional responses also began to emerge during the warning phase. Positive emotional expressions were identified 533 times during this phase and primarily took the form of wishes for safety and concern for people in potentially affected areas. Action behavior primarily involved individual preparedness measures, including sheltering at home, purchasing essential supplies, requesting leave from work, and adjusting travel plans. Supportive behavior remained relatively infrequent. Overall, public responses during the warning phase were characterized by the predominance of information behavior centered on risk verification, accompanied by safety reminders, expressions of concern, and adjustments to everyday routines.

#### 4.1.2. Public Response Behaviors During the Impact Phase

At the coding-reference level, emotional behavior increased markedly during the impact phase, accounting for approximately 73.2% of coded behavioral references, compared with 46.3% during the warning phase, and reaching its highest coded frequency across the three phases (*n* = 4908). At the comment level, emotional behavior was identified in 54.58% of impact-phase comments, compared with 44.36% of warning-phase comments and 50.29% of recovery-phase comments (Table 1). Thus, emotional behavior was descriptively most prevalent during the impact phase at both levels of analysis. This descriptive pattern suggests that the focus of public responses shifted from risk-information acquisition during the warning phase toward immediate experiences of, and emotional reactions to, the disaster’s impacts.

Emotional behavior primarily reflected direct reactions to the immediate impacts of the typhoon. In response to destructive conditions such as strong winds, heavy rainfall, power and network outages, and fallen trees, users frequently expressed fear, concern, and anxiety. These comments were highly immediate and situation-specific, suggesting that social media served as an outlet for the expression and sharing of disaster-related emotional stress.

Notably, emotional responses during the impact phase extended beyond individual fear and anxiety and exhibited a pronounced pattern of cross-regional empathy. Representative comments included “In the end, Yangjiang took it all!” (FS-V0037-C0245), “You took it all for us—it was originally heading toward us” (FS-V0037-C0908), and “People in Zhuhai thank Yangjiang. It was originally going to make landfall in Zhuhai, but then it turned toward Yangjiang” (FS-V0037-C0112). These comments portrayed Yangjiang as the principal affected area that bore the typhoon’s impacts on behalf of other regions. This pattern suggests that users did not interpret the disaster solely in terms of their own exposure; rather, interactions on Douyin produced a geographically differentiated sense of community in which unaffected areas were perceived as having narrowly escaped the disaster while the directly affected area endured its consequences. Such expressions reinforced sympathy and emotional connections among users from different regions while assigning Yangjiang the symbolic role of having borne the disaster on behalf of others.

Although the relative share of information behavior declined during the impact phase, its absolute frequency remained high. Its focus shifted from inquiries about warning information to real-time verification of disaster conditions, as users sought first-hand reports from affected areas. Examples included “Friends in Guangdong, tell us what is happening! Is it getting stronger now?” (FS-V0001-C0010), “How are our friends in Guangdong doing now? It is calm and sunny here in Haikou” (FS-V0011-C0014), and “Reporting from Changan District, Dongguan—the wind is gradually getting stronger. How is it where everyone else is?” (FS-V0009-C0068). These comments demonstrate a clear pattern of information cross-validation. Whereas official meteorological information primarily provided general information on the typhoon’s trajectory, wind intensity, and expected area of impact, user reports from different locations supplied localized and real-time information about actual conditions. By comparing official information with first-hand accounts from other users, the public translated abstract typhoon risk into more concrete understandings of local disaster conditions.

Action behavior primarily consisted of sheltering at home, preparing supplies, watching livestreams, and monitoring surrounding conditions. Supportive behavior included a limited number of responses related to rescue, mutual assistance, and emergency support. Because strong winds and heavy rainfall during landfall restricted outdoor mobility, and on-site rescue required professional expertise and organizational coordination, ordinary users more commonly expressed concern, attention, and gratitude through comments than reported direct offline action. Overall, public responses shifted from the information-dominant pattern observed during the warning phase to a predominantly emotional pattern during the impact phase, accompanied by cross-regional empathy, real-time information verification, and continued monitoring of disaster conditions.

#### 4.1.3. Public Response Behaviors During the Recovery Phase

Emotional behavior remained dominant during the recovery phase. However, its content shifted from fear and concern toward gratitude, encouragement, and expectations of social recovery. A substantial number of comments expressed gratitude and recognition toward rescue personnel, debris-clearance workers, and government emergency-response efforts. Examples included “Salute to all frontline rescuers—you are the real heroes. May you all stay safe” (HF-V0035-C0029), “Thank you to everyone involved in clearing the debris for your hard work” (HF-V0035-C0046), and “Thumbs up to the government!” (HF-V0006-C0001). Meanwhile, comments such as “I hope the typhoon passes soon and things return to normal as quickly as possible” (HF-V0006-C0034) and “Yangjiang will recover” (HF-V0030-C0021) reflected expectations regarding post-disaster reconstruction and the restoration of everyday life. These comments indicate that, as the immediate disaster threat subsided, emotional behavior increasingly took the form of restorative expressions characterized by gratitude, encouragement, and expectations of recovery.

The focus of information behavior also changed during the recovery phase. Rather than primarily seeking information about typhoon intensity and trajectory, users increasingly focused on electricity supply, telecommunications, road clearance, and the restoration of transportation. For example, users commented, “When will electricity be restored to the Shizhu Village Committee area in Dakui, Pinggang Town, Jiangcheng District, Yangjiang? We have been without electricity and internet access for three days” (HF-V0016-C0059); “My internet has been down for several days. When will the broadband service be repaired?” (HF-V0029-C0022); and “The fallen trees in Jiangcheng District have still not been cleared. It will probably take at least several more days” (HF-V0035-C0017). These comments demonstrate that the social consequences of the typhoon persisted after the meteorological event itself had ended. Infrastructure damage and disruptions to public services continued to affect everyday life, and the comment section increasingly served as a space for monitoring recovery progress and articulating practical needs.

The recovery phase also featured a growing number of reflective discussions concerning post-disaster governance and disaster preparedness. Some users proposed improvements related to urban landscaping, tree reinforcement, road clearance, and the organization of personnel. Examples included “Are there better options for selecting urban tree species in coastal cities?” (HF-V0009-C0025), “We should learn from Zhejiang and Fujian and install supports for the trees!” (HF-V0035-C0002), and “District leaders should organize available residents to help clear the roads; each household could send one or two people” (HF-V0035-C0038). Some people also called for continued media attention to recovery efforts, stating, for example, “I hope the media will continue reporting on Yangjiang’s recovery” (HF-V0035-C0052).

Some comments further linked disaster losses to urban planning, preparedness, infrastructure maintenance, and information disclosure. Examples included “There are major problems with site selection and risk assessment in the planning of towns, villages, and housing” (HF-V0012-C0387), “No typhoon-prevention measures were taken before the storm” (HF-V0035-C0041), and “Everyone says it was very serious, so there must have been casualties. I do not understand why they were not reported” (HF-V0035-C0070). Rather than attributing disaster consequences solely to the typhoon itself, these comments questioned the adequacy of pre-disaster preparedness, the timeliness of post-disaster recovery, and the completeness of public information, reflecting an emerging pattern of governance-oriented reflection and structural attribution.

Compared with the preceding two phases, supportive behavior accounted for a somewhat larger proportion of responses during recovery, although its overall frequency remained low. In summary, emotional behavior remained the predominant form of public response during the recovery phase, with restorative emotional expressions becoming increasingly salient. Information behavior, meanwhile, became progressively concentrated on infrastructure restoration, the recovery of public services, and post-disaster governance. Public responses thus shifted from immediate emotional reactions during the disaster toward monitoring recovery, evaluating governance, and reflecting on the structural factors underlying disaster consequences.

### 4.2. Associations of Cognitive Risk Perception and Platform-Interaction Indicators with Public Response Behaviors

Separate binary logistic regression models were estimated for the four response behaviors: information behavior (XX), emotional behavior (QG), action behavior (XD), and supportive behavior (ZC). Each model included cognitive risk perception index (${RP}_{i}$), reply-based conversational engagement (${CE}_{i}$), and like-based social endorsement (${SE}_{i}$), disaster phase, comment exposure duration, Guangdong posting location, and account category. Primary standard errors were clustered by video.

The overall prevalence of each binary outcome is reported in Table 1. Action behavior (73 comments, 0.87%) and supportive behavior (88 comments, 1.05%) were rare. Given these low event counts, Firth logistic regression was additionally used as a sensitivity analysis for these two outcomes. Grouped cross-validation and calibration diagnostics were also conducted to assess the rare-outcome models.

#### 4.2.1. Logistic Regression Results for Information Behavior

The information behavior model was significant (likelihood-ratio *χ*^2^(9) = 1441.138, *p* < 0.001; video-clustered Wald *χ*^2^(9) = 252.697, *p* < 0.001; McFadden R^2^ = 0.133). Coefficient estimates are presented in Table 2.

The cognitive risk perception index was positively associated with information behavior (OR = 1.625, 95% CI [1.482, 1.782], *p* < 0.001), as was reply-based conversational engagement (OR = 1.397, 95% CI [1.268, 1.539], *p* < 0.001). The pooled like-based social endorsement coefficient was not significant (OR = 1.007, *p* = 0.915). Relative to warning-phase comments, information behavior had lower adjusted odds during impact (OR = 0.431, *p* = 0.020) and recovery (OR = 0.190, *p* < 0.001). Exposure duration was negatively associated with information behavior, and Guangdong location was positively associated. Account-type contrasts were not significant. Because phase and exposure duration were strongly aligned, the phase coefficients do not identify independent effects at a common exposure time.

#### 4.2.2. Logistic Regression Results for Emotional Behavior

The emotional behavior model was significant (likelihood-ratio *χ*^2^(9) = 570.733, *p* < 0.001; video-clustered Wald *χ*^2^(9) = 184.224, *p* < 0.001; McFadden R^2^ = 0.049). Coefficient estimates are reported in Table 3.

The cognitive risk perception index was negatively associated with emotional behavior (OR = 0.796, 95% CI [0.728, 0.871], *p* < 0.001), as was reply-based conversational engagement (OR = 0.779, 95% CI [0.704, 0.861], *p* < 0.001). The pooled like-based social endorsement coefficient was not significant (OR = 1.106, *p* = 0.150). Adjusted phase coefficients were not significant; exposure duration was positively associated and Guangdong location negatively associated with emotional behavior.

#### 4.2.3. Logistic Regression Results for Action Behavior

The action behavior model was statistically significant overall (likelihood-ratio *χ*^2^(9) = 43.217, *p* < 0.001; video-clustered Wald *χ*^2^(9) = 54.761, *p* < 0.001; McFadden R^2^ = 0.052). Only 73 action events were observed, so individual coefficients require cautious interpretation. Coefficient estimates are presented in Table 4.

The adjusted ${RP}_{i}$ estimate for action behavior was below 1 but its confidence interval included 1 (OR = 0.753, 95% CI [0.562, 1.008], *p* = 0.057). ${CE}_{i}$ and ${SE}_{i}$ were not significantly associated with action behavior. Guangdong location had a positive association (OR = 1.856, *p* = 0.013). The exposure-duration estimate was very imprecise (OR = 17.864, 95% CI [0.639, 499.278]). In the Firth model, ${RP}_{i}$ also did not reach significance (OR = 0.781, 95% profile CI [0.541, 1.028], *p* = 0.083), whereas Guangdong location remained positively associated (OR = 1.829, *p* = 0.027). The rare outcome, wide exposure interval, and weak validation performance preclude a firm directional inference for ${RP}_{i}$ or prediction of individual action behavior.

#### 4.2.4. Logistic Regression Results for Supportive Behavior

The supportive behavior model was significant overall (likelihood-ratio *χ*^2^(9) = 28.656, *p* < 0.001; video-clustered Wald *χ*^2^(9) = 83.114, *p* < 0.001; McFadden R^2^ = 0.029). Only 88 supportive events were observed, so the estimates are reported with the Firth and calibration analyses. Coefficient estimates are presented in Table 5.

${RP}_{i}$ was not significantly associated with supportive behavior after adjustment (OR = 0.877, 95% CI [0.694, 1.108], *p* = 0.270), nor was ${CE}_{i}$. ${SE}_{i}$ was positively associated in the video-clustered model (OR = 1.370, 95% CI [1.027, 1.826], *p* = 0.032) and in the two-way video/user clustered check (*p* = 0.034). Exposure duration was negatively associated with supportive behavior, and comments under local media and emergency organizations had higher odds than those under national-level authoritative media outlets (OR = 2.385, *p* = 0.002). Firth analysis also found a positive ${SE}_{i}$ association (OR = 1.381, 95% profile CI [1.102, 1.691], *p* = 0.006) but no significant ${RP}_{i}$ association (OR = 0.897, *p* = 0.350). The ${SE}_{i}$ association lost significance when exposure duration was omitted (OR = 1.247, *p* = 0.180), and the rare-outcome model calibrated poorly. This finding should therefore be treated as sensitive to specification, not as firm evidence of a supportive-behavior mechanism.

The video-grouped cross-validation results for the adjusted Firth models are summarized in Table 6.

For action behavior, the video-grouped cross-validated Brier score was 0.00868, compared with a prevalence-only null Brier score of 0.00865; the AUC was 0.590, calibration intercept −0.025, and slope 0.429. For supportive behavior, the corresponding values were 0.01052 versus 0.01041, AUC 0.548, intercept −0.261, and slope 0.203. Both models performed worse than the prevalence-only benchmark on Brier score, discriminated weakly, and yielded overly extreme out-of-fold probabilities; the supportive model also overpredicted on average. They are unsuitable for individual prediction, and their coefficients are interpreted as exploratory associations. Overall tests and fit statistics for the four adjusted logistic regression models are summarized in Table 7.

#### 4.2.5. Rare-Event Sensitivity Analyses

Because action and supportive behaviors were rare, Firth logistic regression with intercept correction was used as a complementary sensitivity analysis. The same focal predictors and controls as in the primary models were retained. Profile penalized-likelihood confidence intervals and *p* values were used consistently. The Firth estimates were not corrected for video-level clustering and therefore do not replace the clustered primary models. The out-of-sample performance of the Firth models was evaluated separately using video-grouped cross-validation. Table 8 presents the adjusted Firth estimates.

In the adjusted Firth models, ${RP}_{i}$ was not statistically significant for action (OR = 0.781, *p* = 0.083) or supportive behavior (OR = 0.897, *p* = 0.350). Guangdong location was positively associated with action behavior. For supportive behavior, ${SE}_{i}$ was positively associated (OR = 1.381, *p* = 0.006), as were the local media and emergency organizations category, while impact phase and exposure duration showed negative associations. ${CE}_{i}$ was not significant in either model. Because these estimates were not cluster-adjusted and predictive performance was weak, they are sensitivity evidence rather than independent confirmation of causal effects.

The Firth models converged under the prespecified numerical controls. Nevertheless, their grouped cross-validated calibration was limited, and neither model improved the Brier score over a prevalence-only predictor. Accordingly, these findings do not justify individual prediction or strong causal claims.

#### 4.2.6. Disaster-Phase Interaction Analyses

To test whether the focal associations varied by disaster phase, adjusted interaction models were estimated for information and emotional behavior. Each model retained the complete set of controls, and inference used video-clustered HC1 covariance estimates. Action and supportive behavior were excluded from the interaction tests because their event counts were too small to support six additional interaction coefficients. Holm adjustment was applied to the six focal two-degree-of-freedom block tests. The Wald tests of the predictor-by-disaster-phase interaction blocks are reported in Table 9.

${RP}_{i}$-by-phase and ${CE}_{i}$-by-phase interactions were nonsignificant for both outcomes. ${SE}_{i}$ varied by phase for information behavior (*χ*^2^(2) = 10.665, Holm-adjusted *p* = 0.024) and emotional behavior (*χ*^2^(2) = 16.671, Holm-adjusted *p* = 0.001). The six emotional interaction coefficients were jointly significant (*χ*^2^(6) = 21.331, *p* = 0.002). These are conditional interaction estimates within the observed posting-time structure. The adjusted phase-specific associations of like-based social endorsement with information and emotional behavior are presented in Table 10.

For information behavior, ${SE}_{i}$ was positively associated during the warning phase (OR = 1.344, 95% CI [1.121, 1.611], *p* = 0.001) but not during impact or recovery. For emotional behavior, ${SE}_{i}$ was positively associated during impact (OR = 1.316, 95% CI [1.117, 1.551], *p* = 0.001) but not during warning or recovery. These within-phase slopes describe heterogeneity in the association; they do not establish an independent causal effect of phase.

#### 4.2.7. Multilevel Sensitivity Analyses

Random-intercept logistic models with comments nested within videos and videos nested within accounts converged without warnings or singularity. The fixed effects were identical to those in the primary models. For information behavior, ${RP}_{i}$ and ${CE}_{i}$ remained positively associated with the outcome, whereas ${SE}_{i}$ remained nonsignificant. For emotional behavior, ${RP}_{i}$ and ${CE}_{i}$ remained negatively associated with the outcome, while ${SE}_{i}$ was positively associated in the multilevel specification. The focal estimates are reported in Table 11.

The total latent-scale clustering ICC was 0.268 for information behavior and 0.261 for emotional behavior; account-level ICCs were 0.015 and 0.002, respectively. Marginal and conditional R^2^ were 0.109 and 0.347 for information behavior and 0.086 and 0.325 for emotional behavior. ${RP}_{i}$ and ${CE}_{i}$ retained their primary-model directions and significance in the multilevel models. Pooled ${SE}_{i}$ remained nonsignificant for information behavior but was positive for emotional behavior in the multilevel model, so the latter main effect is specification-sensitive. In the four primary logistic models, clustering standard errors jointly by video and user did not change the statistical conclusions for the three focal predictors. In models omitting exposure duration, the focal ${RP}_{i}$ and ${CE}_{i}$ conclusions remained, whereas phase estimates changed: the recovery-phase information contrast was no longer significant (OR = 0.582, *p* = 0.369), and action-phase contrasts became significant despite only 73 events. The supportive ${SE}_{i}$ estimate also lost significance (OR = 1.247, *p* = 0.180). Since phase and exposure duration have little common support, phase odds ratios in the main models should be read only as conditional associations. The ${SE}_{i}$-by-phase interactions remained after omission of exposure duration (Holm-adjusted *p* = 0.005 for information and 0.004 for emotional behavior).

### 4.3. Hypothesis Testing Results

H1a was supported because ${RP}_{i}$ was positively associated with information behavior. H1b was not supported because the adjusted ${RP}_{i}$-emotional association was negative. H1c and H1d were not supported because the action and supportive associations did not reach statistical significance in either the video-clustered or Firth models. The rare-event results remain exploratory given weak cross-validated discrimination and calibration.

H2a was partially supported: ${CE}_{i}$ was positively associated with information behavior, but pooled ${SE}_{i}$ was not. H2b was not supported as a stable pooled main-effect hypothesis: ${CE}_{i}$ was negatively associated with emotional behavior, while ${SE}_{i}$ was nonsignificant in the clustered primary model but positive in the multilevel model and during impact in the interaction model. H2c was not supported because neither platform-interaction indicator was significantly associated with action behavior. H2d received limited support because ${SE}_{i}$ was positively associated with supportive behavior in both the clustered and Firth models, whereas ${CE}_{i}$ was not; the supportive outcome was rare and poorly predicted out of sample.

The hypothesis results refer to observable comment-level platform-interaction indicators and a text-based cognitive risk perception index. They should not be generalized to causal effects of individual risk perception or social capital.

## 5. Discussion

The findings provide several insights into public responses to disasters on social media. Response patterns varied across disaster phases and were associated with cognitive risk perception, platform-based interaction, and the practical constraints surrounding offline action.

First, public response behaviors showed distinct patterns across disaster phases, consistent with previous research documenting changes in public attention, emotional reactions, and behavioral responses over the course of disasters. Previous studies have demonstrated that information flows, public attention, and emotional expressions evolve dynamically throughout disaster processes (Hu et al., 2024; Yuan et al., 2021). Research on the “7.20” Henan rainstorm similarly revealed significant temporal variations in public attention, emotional responses, and behavioral expressions (Chen et al., 2025; Wan et al., 2023). In this study, discussions during the warning phase primarily focused on typhoon trajectories, landfall timing, government arrangements, and impacts on work and education, with information behavior representing the dominant response pattern. During this phase, information seeking primarily served the purpose of evaluating typhoon-related risks and adjusting personal arrangements and preparedness strategies accordingly. Thus, the social media comment section functioned not only as a space for risk perception formation but also as a mechanism for coordinating everyday routines before disaster impact.

As the typhoon entered the impact phase, real-time visual content and reports of extreme weather made immediate threats more salient. Emotional behavior was descriptively more prevalent during this phase. Expressions of concern, fear, blessings, and encouragement became prominent during this phase, and comments from users in different regions also conveyed empathy toward affected communities. Meanwhile, information behavior shifted from seeking predictive information about typhoon development during the warning phase toward verifying real-time disaster conditions. By seeking firsthand accounts from affected areas, people supplemented and cross-validated official information through localized observations and experiential reports.

During the recovery phase, as immediate disaster risks gradually declined, emotional expressions shifted from immediate fear, anxiety, and prayers toward restorative emotions, including gratitude toward rescue workers and expectations for the restoration of social and economic activities. Public attention increasingly turned toward infrastructure recovery and broader governance issues, including urban planning, disaster preparedness, and information transparency. This transition suggests that as direct threats diminished, the role of social media users gradually evolved from recipients of disaster information and emotional expressers into evaluators and monitors of post-disaster governance. Therefore, critical discussions during the recovery phase should not be interpreted solely as negative sentiment; they also reflect users’ willingness to participate in post-disaster evaluation and governance feedback through social media.

Second, the cognitive risk perception index showed differentiated associations with the four forms of public response. The index was positively associated with information behavior, indicating that comments articulating more dimensions of likelihood or exposure, vulnerability, and consequences were also more likely to seek, verify, share, or discuss information. This pattern is consistent with research emphasizing that risk appraisal heightens information needs and uncertainty reduction (Yang et al., 2014; Kellens et al., 2013). In contrast, the index was negatively associated with emotional behavior. Because it was deliberately constructed from cognitive risk content and excluded affective expressions, this inverse association should not be read as evidence that greater latent psychological risk perception suppresses emotion. Rather, it suggests that cognitive appraisal and emotional expression constitute partly distinct discursive modes within comments: some comments elaborate exposure and consequences, whereas others communicate fear, concern, sympathy, or encouragement.

The adjusted cognitive risk perception index estimates for action and supportive behavior were imprecise and did not reach statistical significance. Existing research has reported that risk appraisal may encourage preparedness in some settings (Ng, 2022), while high perceived emergency risk can also coincide with lower willingness to engage in rescue activities when participation is costly or hazardous (R. Zhang, 2025). The present study cannot adjudicate those mechanisms because it observes comment content, not offline opportunity or behavior. Action and support likely depend on feasible options, mobility, capacity, resources, and coordinated participation, which were not directly measured here.

Third, the two platform-based interaction indicators showed differentiated associations within the short-video platform environment. Reply-based conversational engagement was positively associated with information behavior but negatively associated with emotional behavior. Comment-based interaction chains provide opportunities for users to ask questions, supplement information, verify facts, and exchange local experiences, and may therefore be more closely related to information-oriented communication. In contrast, emotional expressions do not necessarily require extended conversational exchanges and may be communicated through more immediate and low-cost forms of platform response.

Like-based social endorsement showed model- and phase-dependent patterns. Its pooled coefficients for information and emotional behavior were nonsignificant in the primary video-clustered models. The multilevel model produced a positive pooled association with emotional behavior, whereas the interaction models identified positive associations with information behavior during the warning phase and emotional behavior during the impact phase. Taken together, these findings do not support a single stable main effect. Instead, they suggest that the meaning of liking varies with both model specification and disaster context: endorsement may be attached to useful risk information before landfall and to emotionally resonant expressions during impact. This interpretation is consistent with the broader argument that platform affordances and event contexts shape the social meaning of low-cost engagement signals (Dvir-Gvirsman et al., 2024; Ruan et al., 2022).

Viewed through a social capital perspective, replies and likes are observable forms of interaction on short-video platforms. Replies record exchanges around a comment, whereas likes indicate visible endorsement. Neither indicator directly measures enduring social ties, generalized trust, reciprocity, or access to mobilizable resources. Reply-based conversational engagement was positively associated with information behavior and negatively associated with emotional behavior. Like-based social endorsement was positively associated with supportive behavior in both the video-clustered and Firth models. Phase-specific analyses also found positive associations with information behavior during the warning phase and emotional behavior during the impact phase. The supportive-behavior finding requires caution because only 88 comments were coded for this behavior, cross-validated calibration was weak, and the association was no longer statistically significant when exposure duration was omitted. These results concern supportive expression visible in comments. Whether such expression develops into rescue, volunteering, or material assistance may depend on organizational channels, opportunities for participation, capacity to act, and available resources that this study did not measure.

Overall, the four types of public response showed different descriptive distributions across disaster phases, and their associations with cognitive risk perception and platform interaction also varied. The cognitive risk perception index was positively associated with information behavior and negatively associated with emotional behavior across the main model specifications. The corresponding associations for reply-based conversational engagement were similarly consistent. The pooled association between like-based social endorsement and emotional behavior varied by model specification, while its association with supportive behavior was sensitive to adjustment for exposure duration. Because disaster phase and exposure duration were both derived from comment posting time, the phase coefficients should be interpreted cautiously as model-conditional associations. Action and supportive behavior were infrequent, and the corresponding models showed limited validation performance. These online data therefore provide limited grounds for broader inferences about offline participation.

## 6. Conclusions

Using Typhoon Ragasa in 2025 as a case study, this study analyzed 8365 Douyin comments using qualitative coding, text analysis, and logistic regression. From a disaster-process perspective, it examined how information, emotional, action, and supportive behaviors varied across disaster phases and how the text-based cognitive risk perception index and platform-interaction indicators were associated with each type of behavior. Comparing these behaviors within a single analytical framework provides evidence about public disaster responses in short-video comment sections.

The main findings are summarized as follows. First, public response behaviors demonstrated clear temporal variations throughout the disaster process. During the warning phase, responses were dominated by information seeking and coordination of everyday arrangements; during the impact phase, immediate emotional reactions and cross-regional empathy became prominent; during the recovery phase, responses shifted toward restorative emotions and reflections on disaster governance.

Second, the cognitive risk perception index was positively associated with information behavior and negatively associated with emotional behavior. Its adjusted associations with action and supportive behavior were not statistically significant. The small numbers of action and supportive comments and the models’ weak cross-validated performance limit the conclusions that can be drawn about these two behaviors.

Third, the two platform-interaction indicators showed different patterns. Reply-based conversational engagement was positively associated with information behavior and negatively associated with emotional behavior. Its associations with action and supportive behavior were not statistically significant. Like-based social endorsement was positively associated with supportive behavior in the video-clustered and Firth models, although this association lost significance when exposure duration was omitted. The supportive-behavior model also performed poorly in cross-validation. In the phase-specific analyses, like-based social endorsement was positively associated with information behavior during the warning phase and emotional behavior during the impact phase. These findings suggest that the associations between platform interaction and public response vary across behavioral types and disaster phases.

Through further analysis and discussion, these findings also shed light on the role of short-video platforms in disaster communication. Short-video platforms provide important spaces for information exchange, emotional expression, and social recognition during disasters; however, clear boundaries remain between observable online interaction and more resource-dependent forms of action and support. The relatively low frequencies of action and supportive behaviors may reflect both the practical constraints associated with typhoon events and the limited visibility of offline behaviors in platform comments. Therefore, observable expressions on social media should not be considered equivalent to the full range of public responses occurring offline. Public response behaviors are shaped not only by risk appraisal and online interaction, but also by platform affordances, behavioral costs, organizational conditions, and the availability of real-world resources.

Based on the above findings, three major practical implications can be proposed.

First, differentiated risk communication strategies and public behavior guidance should be implemented according to distinct disaster phases. During the warning phase, relevant authorities should prioritize the provision of clear, specific, and actionable information by linking typhoon trajectories and risk levels with practical measures, such as work and school suspension, evacuation decisions, and household preparedness. Such communication can reduce uncertainty-related anxiety and minimize disruptions to everyday routines. During the impact phase, authorities should continue disseminating authoritative information while enhancing the availability of localized and real-time updates. This approach can address public demands for verification of conditions across different regions and provide a stable informational environment for expressions of fear, concern, and empathy. During the recovery phase, continuous disclosure of information regarding electricity restoration, telecommunications, transportation, public services, and post-disaster response progress is essential. Public concerns, suggestions, and governance evaluations should be incorporated into post-disaster feedback and improvement mechanisms.

Second, the informational and governance feedback functions of social media data should be further leveraged throughout the disaster cycle. Emergency management agencies could employ text analysis, sentiment analysis, and interaction-based indicators to dynamically identify risk-related expressions, information gaps, and frequently discussed concerns. Different response strategies may be adopted for highly commented factual issues and highly endorsed emotional topics. During the recovery phase, comments related to infrastructure, public services, and governance deficiencies can be systematically synthesized, transforming fragmented online opinions into traceable issue lists and actionable governance feedback.

Third, clearer organizational pathways could help people respond to disaster-related needs safely. The cognitive risk perception index was not significantly associated with action or supportive behavior in the adjusted models. Like-based social endorsement was positively associated with supportive comments in the video-clustered and Firth models, although the association lost significance when exposure duration was omitted. These comment-level findings provide limited evidence about actual offline assistance. Government agencies, community organizations, and volunteer groups could publish verified, location-specific information on material needs, donation channels, volunteer opportunities, and safety requirements. During the impact phase, guidance should prioritize sheltering and avoiding hazardous travel. Once conditions are safe, designated organizations could coordinate volunteers and resources according to assessed needs in affected communities.

Although this study systematically identifies the phase-specific characteristics and associated factors of public response behaviors during sudden natural disasters and provides corresponding implications, several limitations remain.

First, regarding the research context, this study focuses on Typhoon Ragasa as a case of sudden natural disaster. However, different hazards, such as typhoons, earthquakes, floods, and rainstorms, vary in terms of onset characteristics, duration, warning conditions, and patterns of public participation. Therefore, the generalizability of the findings requires further examination. Second, regarding measurement, the key explanatory variables were derived from observable textual and platform-interaction traces rather than direct psychological or relational measurements. The cognitive risk perception index reflects cognitive risk expressions in comments, while reply-based conversational engagement and like-based social endorsement capture visible platform interactions. Moreover, because replies and likes may accumulate after a comment is posted, their temporal ordering relative to the coded behavioral expressions cannot be fully established. Therefore, the observed relationships should be interpreted as associations, and reverse directionality cannot be ruled out. Third, the purposive sampling of institutional Douyin accounts was not designed to represent all users or all disaster-related discussions on the platform. Finally, action and supportive behaviors were relatively rare in the sample, with only 73 and 88 events, respectively. Although rare-event sensitivity analyses were conducted, the limited number of cases constrains the precision and generalizability of findings for these two outcomes.

Future research can be extended in four major directions. First, comparative multi-case studies should be conducted. Future studies could compare similarities and differences in public responses across different typhoon events and extend the research context to other sudden natural hazards, including earthquakes, floods, rainstorms, and wildfires. Such comparative analyses would provide deeper insights into the temporal dynamics and associated factors of public response behaviors. Second, social-media data could be combined with surveys or interviews to obtain more direct measures of individuals’ psychological risk perception and social relationships, and to further examine how these variables are related to different forms of public response behavior. Particular attention should be given to the mechanisms through which risk perception influences emotional, supportive and action behaviors, as well as how social capital shapes supportive and action-oriented responses. Third, broader sampling across institutional, self-media, and ordinary-user accounts could provide a more comprehensive picture of public responses on social media. Finally, future research could connect online observations with real-world community settings to trace how action and supportive behaviors emerge and develop beyond platform comments, thereby identifying the social, organizational, and situational factors that facilitate or constrain the transition from online expression to concrete action and support. These efforts would enhance the explanatory power of existing research and contribute to a more comprehensive understanding of public responses during sudden natural disasters.

## Figures and Tables

**Table 1:** Comment-level prevalence of public response behaviors by disaster phase.

Behavior	Warning (*n* = 1472)	Impact (*n* = 5139)	Recovery (*n* = 1754)	Total (*N* = 8365)
Information	719 (48.85%)	1646 (32.03%)	557 (31.76%)	2922 (34.93%)
Emotional	653 (44.36%)	2805 (54.58%)	882 (50.29%)	4340 (51.88%)
Action	29 (1.97%)	38 (0.74%)	6 (0.34%)	73 (0.87%)
Supportive	18 (1.22%)	44 (0.86%)	26 (1.48%)	88 (1.05%)

Note: Values are the number and percentage of comments containing each behavior within the corresponding phase. The denominator is all comments in that phase. Because the four behavioral categories were non-mutually exclusive, percentages are not required to sum to 100%.

**Table 2:** Logistic regression results for information behavior.

Variable	Estimate	Cluster-Robust SE	z-Value	*p*-Value	OR [95% CI]
Intercept	−0.247	0.355	−0.695	0.487	0.781 [0.390, 1.567]
${RP}_{i}$	0.486	0.047	10.344	<0.001 ***	1.625 [1.482, 1.782]
${CE}_{i}$	0.334	0.049	6.785	<0.001 ***	1.397 [1.268, 1.539]
${SE}_{i}$	0.007	0.063	0.107	0.915	1.007 [0.889, 1.140]
Impact phase	−0.843	0.363	−2.319	0.020 *	0.431 [0.211, 0.878]
Recovery phase	−1.661	0.427	−3.892	<0.001 ***	0.190 [0.082, 0.438]
Comment exposure duration	−0.670	0.068	−9.910	<0.001 ***	0.512 [0.448, 0.584]
Guangdong location	0.773	0.174	4.437	<0.001 ***	2.165 [1.539, 3.046]
Government authorities	0.074	0.255	0.290	0.772	1.077 [0.654, 1.773]
Local media and emergency organizations	−0.017	0.278	−0.061	0.952	0.983 [0.570, 1.695]

Note: The warning phase and national-level authoritative media outlets are the reference categories. All continuous predictors are standardized. HC1 standard errors are clustered by video. *** *p* < 0.001, * *p* < 0.05.

**Table 3:** Logistic regression results for emotional behavior.

Variable	Estimate	Cluster-Robust SE	z-Value	*p*-Value	OR [95% CI]
Intercept	−0.077	0.335	−0.231	0.817	0.926 [0.480, 1.785]
${RP}_{i}$	−0.228	0.046	−4.974	<0.001 ***	0.796 [0.728, 0.871]
${CE}_{i}$	−0.250	0.052	−4.850	<0.001 ***	0.779 [0.704, 0.861]
${SE}_{i}$	0.101	0.070	1.441	0.150	1.106 [0.964, 1.268]
Impact phase	0.394	0.335	1.175	0.240	1.483 [0.768, 2.862]
Recovery phase	0.605	0.445	1.358	0.175	1.831 [0.765, 4.383]
Comment exposure duration	0.403	0.071	5.705	<0.001 ***	1.497 [1.303, 1.720]
Guangdong location	−0.471	0.140	−3.359	<0.001 ***	0.624 [0.474, 0.822]
Government authorities	−0.285	0.244	−1.169	0.242	0.752 [0.467, 1.212]
Local media and emergency organizations	0.154	0.271	0.571	0.568	1.167 [0.687, 1.983]

Note: The warning phase and national-level authoritative media outlets are the reference categories. All continuous predictors are standardized. HC1 standard errors are clustered by video. *** *p* < 0.001.

**Table 4:** Logistic regression results for action behavior.

Variable	Estimate	Cluster-Robust SE	z-Value	*p*-Value	OR [95% CI]
Intercept	−5.752	0.876	−6.566	<0.001 ***	0.003 [0.001, 0.018]
${RP}_{i}$	−0.284	0.149	−1.905	0.057	0.753 [0.562, 1.008]
${CE}_{i}$	−0.165	0.145	−1.137	0.255	0.848 [0.638, 1.127]
${SE}_{i}$	−0.133	0.134	−0.991	0.322	0.876 [0.673, 1.139]
Impact phase	−0.353	0.509	−0.693	0.488	0.703 [0.259, 1.906]
Recovery phase	−0.014	0.933	−0.015	0.988	0.986 [0.159, 6.137]
Comment exposure duration	2.883	1.699	1.697	0.090	17.864 [0.639, 499.278]
Guangdong location	0.618	0.249	2.481	0.013 *	1.856 [1.139, 3.025]
Government authorities	0.250	0.472	0.531	0.596	1.285 [0.510, 3.238]
Local media and emergency organizations	0.148	0.446	0.331	0.740	1.159 [0.484, 2.777]

Note: The warning phase and national-level authoritative media outlets are the reference categories. All continuous predictors are standardized. HC1 standard errors are clustered by video. *** *p* < 0.001, * *p* < 0.05.

**Table 5:** Logistic regression results for supportive behavior.

Variable	Estimate	Cluster-Robust SE	z-Value	*p*-Value	OR [95% CI]
Intercept	−4.375	0.358	−12.218	<0.001 ***	0.013 [0.006, 0.025]
${RP}_{i}$	−0.131	0.119	−1.103	0.270	0.877 [0.694, 1.108]
${CE}_{i}$	0.007	0.116	0.065	0.948	1.008 [0.803, 1.264]
${SE}_{i}$	0.314	0.147	2.141	0.032 *	1.370 [1.027, 1.826]
Impact phase	−0.626	0.381	−1.643	0.100	0.535 [0.254, 1.128]
Recovery phase	−0.627	0.455	−1.379	0.168	0.534 [0.219, 1.302]
Comment exposure duration	−0.353	0.083	−4.234	<0.001 ***	0.702 [0.596, 0.827]
Guangdong location	−0.153	0.246	−0.621	0.535	0.859 [0.530, 1.390]
Government authorities	−0.260	0.600	−0.433	0.665	0.771 [0.238, 2.500]
Local media and emergency organizations	0.869	0.285	3.047	0.002 **	2.385 [1.364, 4.172]

Note: The warning phase and national-level authoritative media outlets are the reference categories. All continuous predictors are standardized. HC1 standard errors are clustered by video. *** *p* < 0.001, ** *p* < 0.01, * *p* < 0.05.

**Table 6:** Video-grouped 10-fold cross-validation of adjusted Firth models for action and supportive behavior.

Outcome	Events	Brier	Null Brier	AUC	Calibration Intercept/Slope
Action	73	0.00868	0.00865	0.590	−0.025/0.429
Supportive	88	0.01052	0.01041	0.548	−0.261/0.203

**Table 7:** Overall tests and fit statistics for the four adjusted logistic regression models.

Model	LR *χ*^2^(9)	LR *p*	Robust Wald *χ*^2^(9)	Robust Wald *p*	AIC
Information behavior	1441.138	<0.001	252.697	<0.001	9403.508
Emotional behavior	570.733	<0.001	184.224	<0.001	11,033.755
Action behavior	43.217	<0.001	54.761	<0.001	814.381
Supportive behavior	28.656	<0.001	83.114	<0.001	968.003

Note: All models included 8365 comments, 88 video clusters, three focal predictors, and six covariate coefficients (two phase, two account-type, one location, and one exposure-duration coefficient). LR = likelihood ratio.

**Table 8:** Adjusted Firth penalized-likelihood logistic regression for action and supportive behaviors.

Predictor	Action B	Action OR [95% CI]	*p*	Supportive B	Supportive OR [95% CI]	*p*
Intercept	−4.623	0.010 [0.005, 0.021]	<0.001	−4.373	0.013 [0.007, 0.023]	<0.001
${RP}_{i}$	−0.247	0.781 [0.541, 1.028]	0.083	−0.109	0.897 [0.682, 1.113]	0.350
${CE}_{i}$	−0.136	0.873 [0.615, 1.166]	0.380	0.015	1.015 [0.819, 1.231]	0.883
${SE}_{i}$	−0.117	0.889 [0.625, 1.208]	0.474	0.322	1.381 [1.102, 1.691]	0.006
Impact phase	−0.812	0.444 [0.257, 1.625]	0.621	−0.641	0.527 [0.300, 0.962]	0.037
Recovery phase	−1.083	0.339 [0.107, 6.313]	1.000	−0.622	0.537 [0.242, 1.161]	0.114
Comment exposure duration	0.537	1.711 [0.869, 507.434]	0.161	−0.354	0.702 [0.577, 0.858]	<0.001
Guangdong location	0.604	1.829 [1.072, 3.182]	0.027	−0.155	0.857 [0.515, 1.423]	0.550
Government authorities	0.219	1.245 [0.602, 2.464]	0.544	−0.186	0.830 [0.284, 2.050]	0.703
Local media and emergency organizations	−0.049	0.953 [0.516, 2.339]	0.877	0.868	2.382 [1.323, 4.335]	0.004

Note: B denotes the estimated logit coefficient. The warning phase and national-level authoritative media outlets are the reference categories. Continuous predictors are standardized. Confidence intervals and *p* values use profile penalized-likelihood inference. These rare-outcome estimates are not adjusted for video clustering.

**Table 9:** Wald tests of predictor-by-disaster-phase interactions.

Outcome	Interaction Block	Wald χ^2^	df	*p*	Holm *p*
Information behavior	${RP}_{i}$ × phase	4.593	2	0.101	0.402
Information behavior	${CE}_{i}$ × phase	0.078	2	0.962	1.000
Information behavior	${SE}_{i}$ × phase	10.665	2	0.005	0.024
Emotional behavior	${RP}_{i}$ × phase	0.122	2	0.941	1.000
Emotional behavior	${CE}_{i}$ × phase	0.563	2	0.755	1.000
Emotional behavior	${SE}_{i}$ × phase	16.671	2	<0.001	0.001
Emotional behavior	All six interactions	21.331	6	0.002	—

Note: Wald χ^2^ tests use two degrees of freedom for each predictor block; the emotional omnibus test uses six degrees of freedom.

**Table 10:** Adjusted phase-specific associations of like-based social endorsement with information and emotional behavior.

Outcome	Phase	B	Robust SE	OR [95% CI]	*p*
Information behavior	Warning	0.296	0.092	1.344 [1.121, 1.611]	0.001
Information behavior	Impact	−0.096	0.085	0.908 [0.768, 1.074]	0.259
Information behavior	Recovery	−0.025	0.212	0.975 [0.644, 1.477]	0.906
Emotional behavior	Warning	−0.165	0.089	0.848 [0.712, 1.009]	0.064
Emotional behavior	Impact	0.275	0.084	1.316 [1.117, 1.551]	0.001
Emotional behavior	Recovery	−0.177	0.161	0.838 [0.611, 1.150]	0.274

Note: B denotes the estimated logit coefficient. Estimates derive from fully adjusted interaction models with video-clustered HC1 standard errors.

**Table 11:** Focal estimates from multilevel random-intercept sensitivity models.

Outcome	Predictor	B	SE	OR [95% CI]	*p*
Information	${RP}_{i}$	0.441	0.027	1.555 [1.476, 1.637]	<0.001
Information	${CE}_{i}$	0.309	0.030	1.362 [1.285, 1.444]	<0.001
Information	${SE}_{i}$	0.047	0.037	1.048 [0.976, 1.126]	0.199
Emotional	${RP}_{i}$	−0.228	0.025	0.796 [0.757, 0.836]	<0.001
Emotional	${CE}_{i}$	−0.276	0.029	0.758 [0.716, 0.803]	<0.001
Emotional	${SE}_{i}$	0.153	0.034	1.165 [1.090, 1.245]	<0.001

Note: B denotes the estimated logit coefficient. Models include random intercepts for account and video and retain the same fixed effects and controls as the primary models. *N* = 8365 comments, 88 videos, and 10 accounts. Continuous predictors are standardized. These estimates are sensitivity analyses and do not replace the video-clustered primary inference.

## Data Availability

The data presented in this study are available on request from the first author.
